# Reconstruction of the complete mitogenomes of predator and prey from a faecal metagenomic dataset

**DOI:** 10.1016/j.dib.2023.109830

**Published:** 2023-11-20

**Authors:** Arsalan Emami-Khoyi

**Affiliations:** Centre for Ecological Genomics and Wildlife Conservation, University of Johannesburg, Auckland Park 2006, South Africa

**Keywords:** Non-invasive methods, Environmental DNA, Faecal metagenomics, Mitogenome, Estuarine pipefish

## Abstract

The application of faecal DNA in genetic studies of wild populations minimises disturbances to their normal behaviours and body integrity. Here, I present an analysis of a metagenomic dataset generated from the faecal DNA of several specimens of the estuarine pipefish, *Syngnathus watermeyeri,* to simultaneously assemble the mitogenomes of the predator and its main prey species, the copepod *Pseudodiaptomus hessei*. The mitogenomes of the pipefish and the copepod were successfully reconstructed using a combination of short seed extension and denovo metagenomic assembly. Nucleotide blast searches of the circular contigs, mitogenome annotations, and Bayesian phylogenetic analyses confirm the completeness and correct taxonomic placements of the two mitogenomes. In addition, heteroplasmy detection and Pool-Seq variant calling quantified the level of genetic diversity in the sequences that formed these assemblies. These can be used as a first step to non-invasively survey genetic diversity in these populations.

Specifications Table

**Table:** 

Subject	Genomics
Specific subject area	Mitogenomics, Critically Endangered estuarine pipefish, *Syngnathus watermeyeri*, Bayesian phylogenetics, non-invasive methods, faecal metagenomic.
Data format	Raw and Analyzed
Type of data	Figures: Mitogenome circular map, phylogenetic tree, single nucleotide polymorphic sites tree map. Table: List of Syngnathidae mitogenomes used for phylogenetic study, List of copepod mitogenomes used for phylogenetic study Mitogenome annotation feature table. FASTA: Mitogenome sequence data
Data collection	Tissue collection: Faecal sample Genomic DNA extraction: Qiagen DNeasy Power Soil Pro (Qiagen, Hilden, Germany), Standard CTAB protocol, NucleoSpin (Macherey-Nagel, Dueren, Germany). Genomic library preparation: Diagenode Bioruptor (Diagenode Bioruptor, New Jersey, United States), NOVOKit (Novogene, Beijing, PRC), NovaSeq 6000 SP platform (Illumina, San Diego, USA). Quality check: FastQC v.0.12, Trimmomatic v.3.9. Mitogenome assembly, annotation and variant calling: NOVOPlasty v.4.3, GetOrganelle v.1.7, MITOS web server, MitoZ v.3.6, Freebayes v.1.3, samtools v.1.6 Phylogenetic analysis: BEAST2 v.2.5, RB package, Tracer 1.7. Phylogenetic tree visualisation: Figtree v.1.4, Chloroplot v.0.2, strap v.1.6
Data source location	Institute: University of Johannesburg City: Johannesburg Province: Gauteng Country: South Africa Latitude and longitude of the specimen collection site: -33.67378 south, 26.67484 east email: molzoo@uj.ac.za
Data accessibility	Repository name: NCBI nucleotide database Data identification number: OR601689 Direct URL to data: https://www.ncbi.nlm.nih.gov/nuccore/OR601689.1/ Repository name: NCBI BioProject Data identification number: PRJNA911940 Direct URL to data: https://www.ncbi.nlm.nih.gov/bioproject/?term=PRJNA911940 Repository name: NCBI BioSample Data identification number: SAMN32216042 Direct URL to data: https://www.ncbi.nlm.nih.gov/biosample/32216042 Repository name: Sequence Read Archive Data identification number: SRX18688242 Direct URL to data: https://trace.ncbi.nlm.nih.gov/Traces/?view=run_browser&acc=SRR22726721&display=metadata
Related research article	https://www.frontiersin.org/articles/10.3389/fmars.2023.1116741/full

## 1 Value of the Data

• The analysed metagenomic dataset is the first to simultaneously assemble the mitogenomes of a predator and its major prey species from non-invasively sampled faecal DNA.
• It provides researchers with a new method to acquire genetic data from wild species without the need to immobilise or even directly observe them in the field. It can also be used as a first step in non-invasively assessing genetic diversity in wild populations.
• It indicates that a large amount of unexplored mitogenomic sequencing data that are already available in public genomic databases can potentially contribute to the development of more extensive reference databases for genetic studies.

## 2 Background

Genetic studies on wild populations have traditionally relied on samples that were collected intrusively, e.g., through lethal methods or by taking tissue samples from animals that were immobilised physically or chemically [1].

Even when non-lethal methods are used, survival is not guaranteed because the target animals are subjected to significant stress, and the small injuries resulting from the acquisition of tissue samples may result in excessive bleeding and serious infection [2,3]. The application of intrusive sampling methods in the study of small-bodied, rare, and threatened species is thus potentially problematic.

With the recent development of more sensitive biochemical assays and sequencing technologies, coupled with high-throughput computational resources, it is now possible to obtain a comparable level of information by sequencing the trace amounts of DNA that animals leave behind in their surrounding environments (environmental DNA, or eDNA), and thus minimise the level of stress or injury to which the subjects are exposed [4].

Molecular reconstruction of diet by high-throughput sequencing of DNA retrieved from body excretions, such as faeces or regurgitates, provides valuable information about a species’ dietary requirements across a broader taxonomic range and on a shorter time scale than morphological identification of hard part contents of gut or faecal samples [5]. However, exposure to digestion enzymes in the alimentary canal fragments DNA into shorter pieces, making the sequencing of the relatively long regions that are typically targeted by PCR-based approaches a challenging task [6]. This has necessitated the use of diagnostic short fragments of gene regions from the mitochondrial genome to identify prey species [7]. While these markers are useful for distinguishing between different taxa at higher taxonomic levels, longer DNA fragments or sequencing the complete mitogenome are usually needed to conclusively resolve taxonomic and phylogenetic uncertainties [8].

As the output of sequencing platforms increases, the application of faecal metagenomic DNA for simultaneous assembly not only of the complete mitogenomes of a predator but also of the prey species it has consumed has become feasible, but it remains largely unexplored [8,9].

The Critically Endangered South African estuarine pipefish, *Syngnathus watermeyeri* Smith, 1963 is one of the rarest fish species on Earth. In 1994, it was declared extinct by the IUCN until a small population was rediscovered in 2006 [10]. Its current distribution range is restricted to narrow strips of submerged macrophytes (mainly *Zostera capensis* and *Ruppia cirrhosa*) in the Kariega and Bushmans estuaries on the south-eastern coast of South Africa. Its catastrophic population decline has been attributed to poorly managed coastal development plans that resulted in the degradation of its estuarine habitats [11]. Similar to other species of pipefish, *S. watermeyeri* is characterised by low mobility, small home ranges and low fecundity, which can negatively impact the successful implementation of conservation plans [11]. Thus, genomic resources for this species are needed to establish a multifaceted effective conservation plan to protect its last remaining populations.

In this analysed dataset, I evaluated whether it is possible to use random shotgun sequencing of faecal metagenomic DNA to simultaneously assemble the complete mitogenome of *Syngnathus watermeyeri* and that of its most important prey species, the copepod *Pseudodiaptomus hessei* (Mrázek, 1894).

## 3 Data Description

**Fig. 1:**
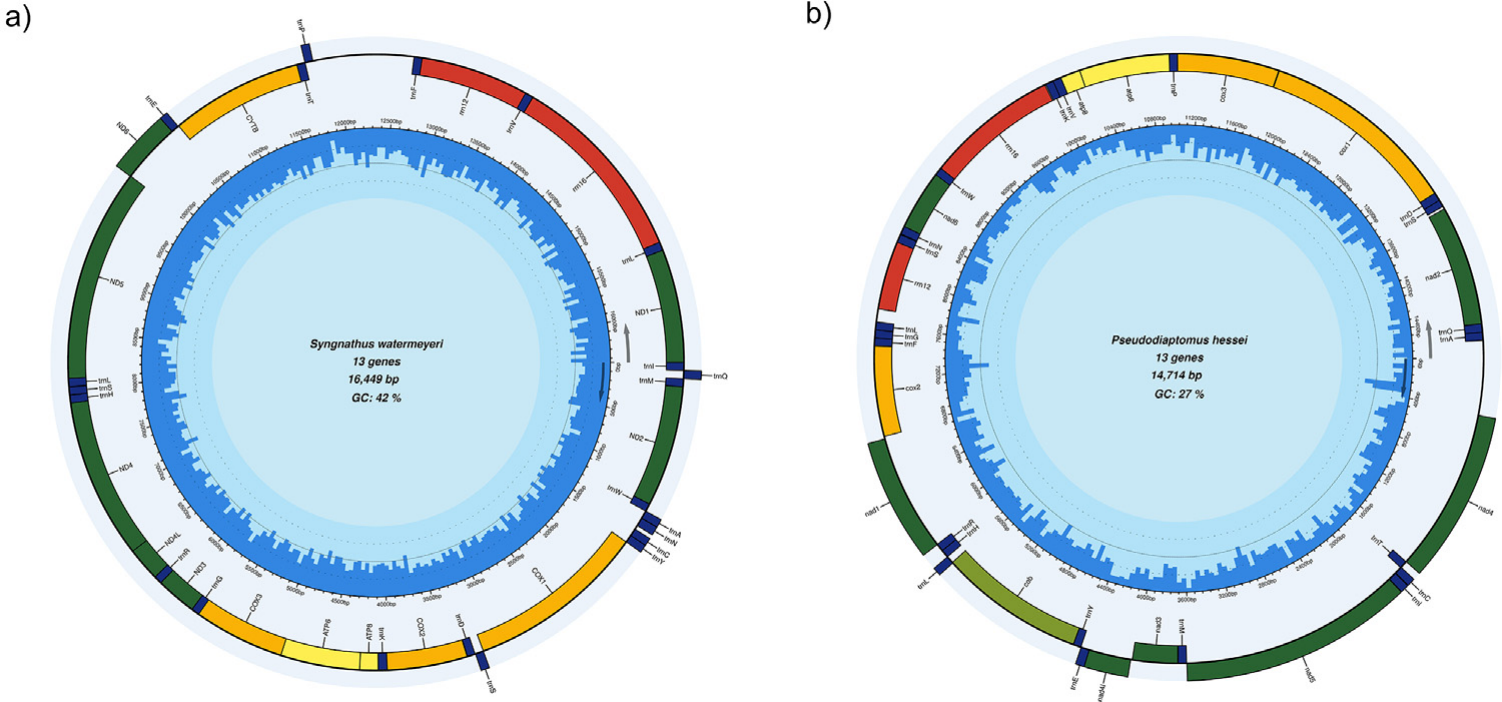
Circular representations of the mitogenomes of (a) the pipefish *Syngnathus watermeyeri* and (b) the copepod *Pseudodiaptomus hessei* assembled from pipefish faecal metagenomic DNA. The positions of the protein-coding genes, tRNAs, and rRNAs are shown in different colours. The interior blue bars show the GC content.

An Illumina sequencing run yielded a total of 35,293,150 paired-end sequences. The NOVOPlasty pipeline successfully retrieved the estuarine pipefish initial *cytb* seed from pooled faecal DNA originating from several pipefishes, and a circular contig 16,449 bp in length was assembled (Fig. 1a). A blast search against the NCBI database identified the only published mitogenome of *Syngnathus watermeyeri*, which was assembled from DNA extracted from a fin clip biopsy (NCBI accession number OR496150), as the closest match, with 99.9 % identity and 100 % coverage. When this assembly was used as the guiding reference template to re-assemble the complete mitogenome denovo in the GetOrganelle toolkit, an identical circular contig was retrieved, confirming the completeness of the assembly.

The MitoZ pipeline assembled a circular contig 14,714 bp long, with 75.69 % identity to the mitogenome of the copepod *Phyllodiaptomus tunguidus* (NCBI accession number MW971444.1), a species that does not occur in South Africa (Fig. 1b). However, when the annotated *COI* sequence from this contig was searched against the nucleotide database, it was 99.52 % identical to the *COI* gene sequence from *Pseudodiaptomus hessei* (NCBI accession number OM747860.1), a species that was recently identified as the major prey item of the estuarine pipefish based on *COI* metabarcoding [12]. In addition, a neighbour-joining phylogenetic tree that was constructed based on the annotated *COI* sequence of the assembled mitogenome, *COI* sequences from previously published records of the same species in the same geographic region, and other closely related calanoid copepods confirmed that sequences from the assembled mitogenome clustered with other *COI* sequences generated from *Pseudodiaptomus hessei* tissue samples with 100 % bootstrap support (Supplementary Information, Fig. 1).

**Fig. 2:**
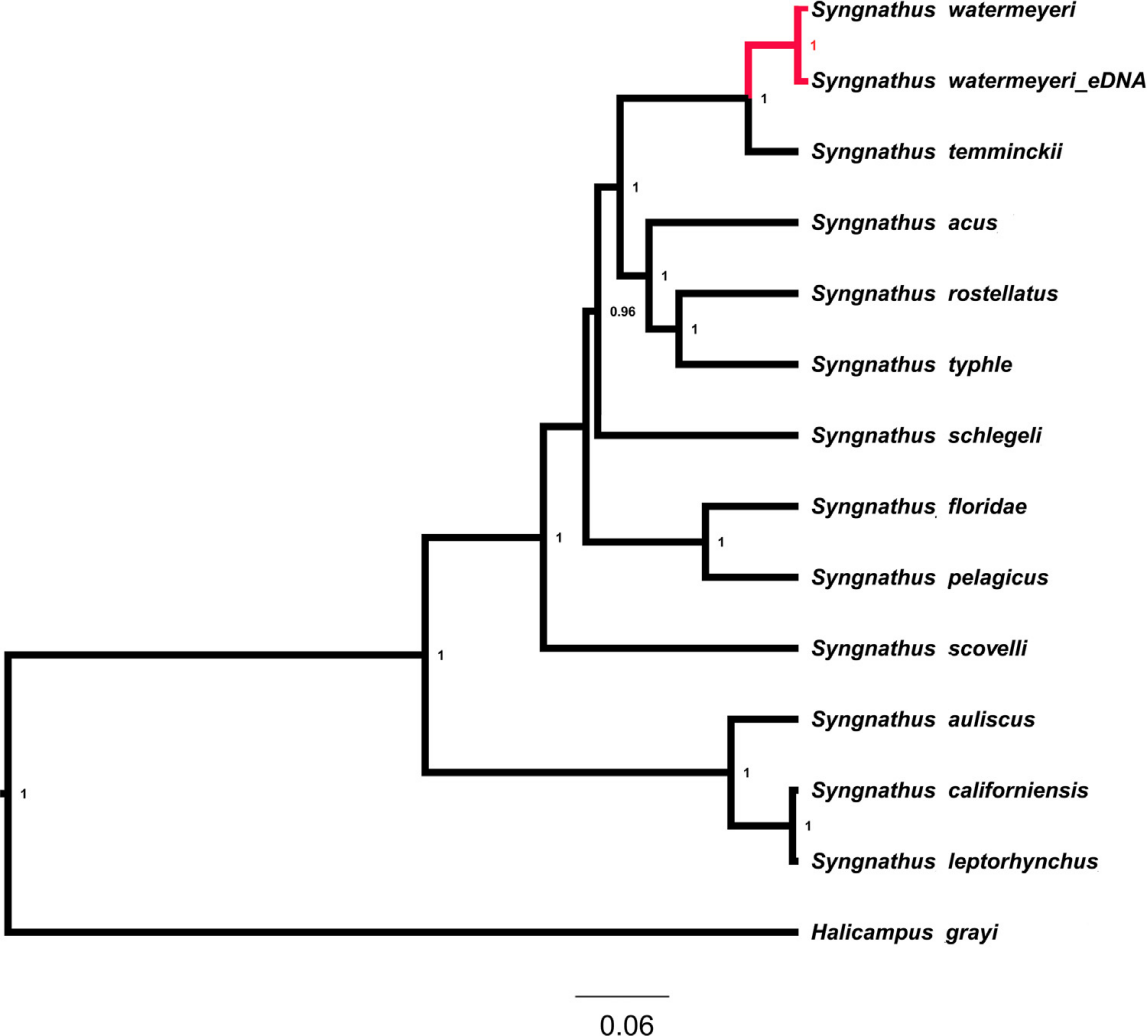
Bayesian phylogenetic tree showing the phylogenetic placement of the mitogenomes of *Syngnathus watermeyeri* among other species of pipefish. The numbers next to nodes represent posterior probabilities. The branches leading to the two assemblies are shown in red, with “*Syngnathus watermeyeri*_eDNA” representing the mitogenome reconstructed in the present study and “*Syngnathus watermeyeri*” representing a previously reconstructed mitogenome using a fin clip biopsy. The scale bar is proportional to the number of substitutions in each branch.

**Fig. 3:**
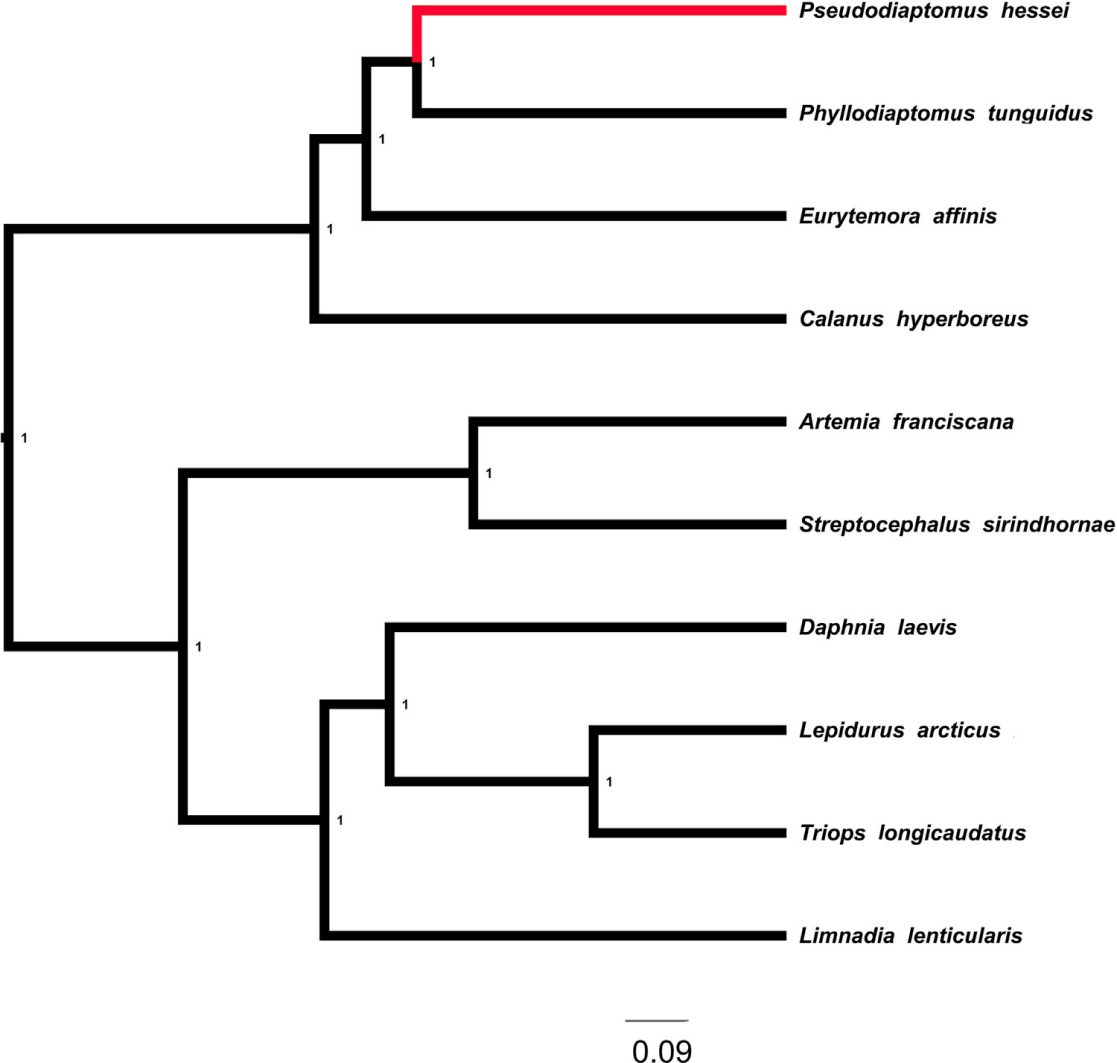
Bayesian phylogenetic tree showing the phylogenetic placement of the mitogenome of Pseudodiaptomus hessei among other crustaceans. The numbers next to nodes represent posterior probabilities. The branches leading to the two species are shown in red. The scale bar is proportional to the number of substitutions in each branch.

A total of 37 mitogenomic features, including 13 protein-coding genes, 22 tRNAs and 2 rRNAs, were annotated on two assembled contigs (Tables 1 and 2). Comparative Bayesian phylogenetic analysis (Table 3) shows that the pipefish mitogenome that was assembled from faecal metagenomic DNA clusters with that assembled from tissue sample with a posterior probability of 1. These two almost identical mitogenomes form a monophyletic group with another southern African pipefish species, *Syngnathus temminckii*, and this clade of southern African pipefishes has a sister taxon relationship with a clade of northern hemisphere pipefishes that includes *S. acus, S. rostellatus,* and *S. typhle* (Fig. 2). The mitogenome of *P. hessei* is the first to be assembled for this species. In the phylogenetic tree (Fig. 3), it clusters with other members of the copepod order Calanoida with a posterior probability of 1, and their phylogenetic placement relative to the Notostraca (*Triops longicaudatus, Lepidurus arcticus*), Artemidae, Streptocephalidae, Limnadidae and Daphniidae was correctly recovered.

**Table 1:** A description of the 37 annotated mitogenomic features in the mitogenome of*Syngnathus watermeyeri.*

Feature	Start	End	Strand	Product
tRNA	1	68	+	tRNA-Ile
tRNA	69	138	-	tRNA-Gln
tRNA	140	208	+	tRNA-Met
CDS	209	1250	+	NADH dehydrogenase subunit 2
tRNA	1251	1318	+	tRNA-Trp
tRNA	1319	1387	-	tRNA-Ala
tRNA	1389	1461	-	tRNA-Asn
tRNA	1497	1561	-	tRNA-Cys
tRNA	1562	1628	-	tRNA-Tyr
CDS	1630	3180	+	cytochrome c oxidase subunit I
tRNA	3181	3251	-	tRNA-Ser
tRNA	3255	3322	+	tRNA-Asp
CDS	3326	4016	+	cytochrome c oxidase subunit II
tRNA	4017	4092	+	tRNA-Lys
CDS	4094	4261	+	ATP synthase F0 subunit 8
CDS	4252	4935	+	ATP synthase F0 subunit 6
CDS	4935	5719	+	cytochrome c oxidase subunit III
tRNA	5719	5788	+	tRNA-Gly
CDS	5789	6137	+	NADH dehydrogenase subunit 3
tRNA	6138	6206	+	tRNA-Arg
CDS	6207	6503	+	NADH dehydrogenase subunit 4L
CDS	6497	7877	+	NADH dehydrogenase subunit 4
tRNA	7878	7946	+	tRNA-His
tRNA	7947	8014	+	tRNA-Ser
tRNA	8018	8088	+	tRNA-Leu
CDS	8089	9924	+	NADH dehydrogenase subunit 5
CDS	9921	10442	-	NADH dehydrogenase subunit 6
tRNA	10443	10510	-	tRNA-Glu
CDS	10516	11656	+	cytochrome b
tRNA	11657	11728	+	tRNA-Thr
tRNA	11728	11796	-	tRNA-Pro
tRNA	12662	12731	+	tRNA-Phe
rRNA	12732	13664	+	12S ribosomal RNA
tRNA	13664	13735	+	tRNA-Val
rRNA	13737	15399	+	16S ribosomal RNA
tRNA	15400	15472	+	tRNA-Leu
CDS	15473	16447	+	NADH dehydrogenase subunit 1

**Table 2:** A description of the 37 annotated mitogenomic features in the mitogenome of *Pseudodiaptomus hessei* assembled from faecal metagenomic DNA of the pipefish *Syngnathus watermeyeri*.

Feature	Start	End	Strand	Product
CDS	439	1707	-	NADH dehydrogenase subunit 4
tRNA	1709	1770	+	tRNA-Thr
tRNA	1766	1826	-	tRNA-Cys
tRNA	1830	1893	-	tRNA-Ile
CDS	1896	3617	-	NADH dehydrogenase subunit 5
tRNA	3620	3684	+	tRNA-Met
CDS	3688	4038	+	NADH dehydrogenase subunit 3
CDS	4051	4377	-	NADH dehydrogenase subunit 4L
tRNA	4378	4443	-	tRNA-Glu
tRNA	4446	4506	+	tRNA-Tyr
CDS	4508	5641	+	cytochrome b
tRNA	5636	5698	-	tRNA-Leu
tRNA	5699	5760	+	tRNA-His
tRNA	5761	5822	+	tRNA-Arg
CDS	5821	6732	-	NADH dehydrogenase subunit 1
CDS	6752	7454	+	cytochrome c oxidase subunit 2
tRNA	7455	7515	+	tRNA-Phe
tRNA	7516	7577	+	tRNA-Gly
tRNA	7578	7639	+	tRNA-Leu
rRNA	7741	8284	+	12S ribosomal RNA
tRNA	8274	8338	+	tRNA-Ser
tRNA	8344	8406	+	tRNA-Asn
CDS	8407	8874	+	NADH dehydrogenase subunit 6
tRNA	8873	8935	+	tRNA-Trp
rRNA	8936	9968	+	16S ribosomal RNA
tRNA	9977	10038	+	tRNA-Lys
tRNA	10038	10102	+	tRNA-Val
CDS	10103	10264	+	ATP synthase F0 subunit 8
CDS	10258	10960	+	ATP synthase F0 subunit 6
tRNA	10961	11023	+	tRNA-Pro
CDS	11026	11817	+	cytochrome c oxidase subunit 3
CDS	11822	13370	+	cytochrome c oxidase subunit 1
tRNA	13371	13437	+	tRNA-Asp
tRNA	13436	13490	+	tRNA-Ser
CDS	13512	14445	+	NADH dehydrogenase subunit 2
tRNA	14446	14511	+	tRNA-Gln
tRNA	14512	14574	+	tRNA-Ala

**Table 3:** Names and NCBI accession numbers of the species used to reconstruct Bayesian phylogenetic trees for *Syngnathus watermeyeri* and *Pseudodiaptomus hessei*.

Species	Accession number
*Syngnathus rostellatus*	MN122827.1
*Hippocampus trimaculatus*	NC_021107.1
*Hippocampus barbouri*	NC_024536.1
*Hippocampus comes*	NC_020336.1
*Syngnathus typhle*	MN122884.1
*Hippocampus abdominalis*	NC_028181.1
*Syngnathus pelagicus*	NC_065498.1
*Syngnathus auliscus*	OL334979.1
*Syngnathus californiensis*	NC_063774.1
*Syngnathus leptorhynchus*	NC_063777.1
*Syngnathus floridae*	NC_065497.1
*Syngnathus scovelli*	NC_065499.1
*Syngnathus acus*	MN122937.1
*Syngnathus schlegeli*	NC_037520.1
*Pseudodiaptomus hessei*	OR601689
*Phyllodiaptomus tunguidus*	NC_046743.1
*Eurytemora affinis*	NC_046694.1
*Daphnia laevis*	NC_045243.1
*Lepidurus arcticus*	NC_044654.1
*Limnadia lenticularis*	NC_039394.1
*Calanus hyperboreus*	NC_019627.1
*Triops longicaudatus*	NC_006079.1
*Artemia franciscana*	MT495440.1
*Streptocephalus sirindhornae*	KP273593.1
*Syngnathus watermeyeri*	OR496150.1
*Phoxocampus belcheri*	NC_065495.1

The successful assembly of two circular contigs, complete annotation of 37 mitogenomic features on both mitogenomes, and the correct phylogenetic placement of the two species confirmed that the complete mitogenomes of the predator and its main prey item were successfully assembled from the faecal metagenomic dataset.

**Fig. 4:**
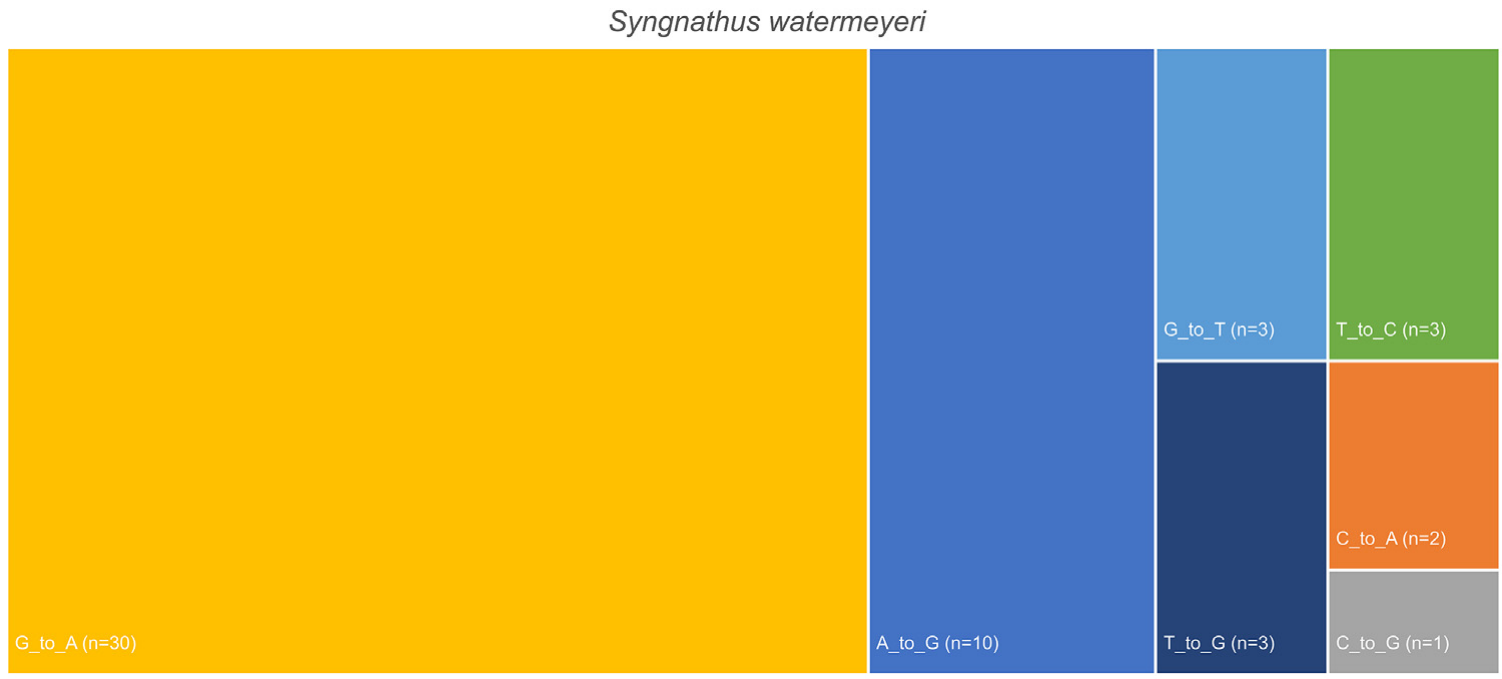
Number single nucleotide polymorphic sites (SNPs) identified from metagenomic sequences that were used to assemble the mitochondrial genome of *Syngnathus watermeyeri*.

**Fig. 5:**
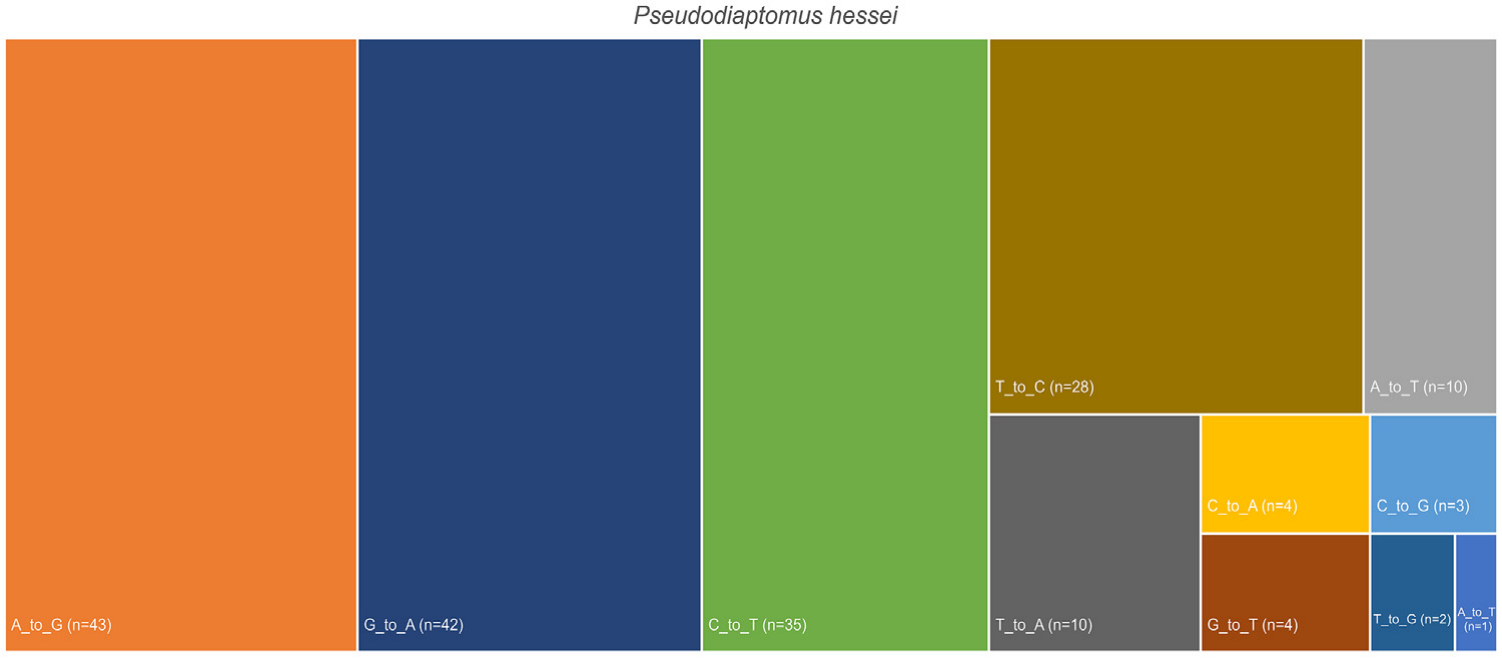
Number single nucleotide polymorphic sites (SNPs) identified from metagenomic sequences that were used to assemble the mitochondrial genome of the copepod *Pseudodiaptomus hessei.*

Heteroplasmy detection identified a total of 62 putative heteroplasmic variable sites in the pipefish mitogenome (Fig. 4). Similarly, when the raw sequences were mapped against the assembled copepod mitogenome, a total of 211 SNPs with an average allele count of 2.08 were assigned (Fig. 5). These results reflect the level of mitochondrial genetic diversity in the faecal samples for the predator and its major prey, and the putative number of alleles in each assembly can serve as preliminary estimates of minimum genetic diversity in the populations of both the predator and its prey (Fig. 5).

## 4 Experimental Design, Materials and Methods

Five pipefish were caught with a seine net in the Bushmans Estuary on the southeast coast of South Africa (-33.682215 south, 26.640596 east). They were immediately transferred to a well-aerated aquarium containing estuarine water from the collection site, which was partly replaced every 30 min. The pipefish were closely monitored for three hours before releasing them back to the site in the estuary where they had been captured. All faecal pellets that they had dropped in the aquarium during this time were then collected using sterile tweezers and transferred into an Eppendorf tube containing 99 % ethanol. Faecal DNA was extracted using the standard CTAB protocol [13], DNeasy Power Soil Pro (Qiagen, Hilden, Germany) and NucleoSpin (Macherey-Nagel, Dueren, Germany). Based on agarose gel visualisation, the three extraction methods produced metagenomic DNA of similar quality, and these extractions were pooled before genomic library preparation. To construct a genomic library, the extracted DNA was first fragmented using a Diagenode Bioruptor (Diagenode, New Jersy, United States), end-repaired, and adapter-ligated as explained in the NEB ‘s kit instruction (NEB, Massachusetts, USA) and only DNA fragments ∼350 bp in length were selected for the amplification step. The quality of the genomic library was inspected using Qubit (Thermo Fisher Scientific, Waltham, USA), qPCR, and the DNA NGS 3K assay (PerkinElmer, Waltham, USA). The generated library was sequenced on the NovaSeq 6000 SP platform (Illumina, San Diego, USA) using paired-end 150 bp chemistry.

The quality of the resulting sequences and the presence of adapter contaminations were checked in FastQC v0.12 (https://www.bioinformatics.babraham.ac.uk/projects/fastqc/), and potential adapter contaminations and low-quality sequences, which were defined as sequences that contain 4 consecutive base pairs with an average Phred score quality below 20 were removed using Trimmomatic v.0.39 [14].

I followed two different methods to assemble mitogenomes: a seed extension and a k-mer based denovo cross-validation approach for the pipefish and a k-mer based denovo metagenomic assembly for its prey. The DNA of a predator is significantly more abundant in the faeces than that of the prey it has consumed [15,16]. This suggests that while mitochondrial seed initiation is more feasible for the predator, denovo assembly is preferable for the prey. The pipefish mitogenome was assembled by the extension of a *cytb* seed sequence from the same species (NCBI accession number JX228139.1) in NOVOPlasty v4.3 [17]. Then, this assembled circular contig was used as the guiding reference template in GetOrganelle v.1.7 [18] to verify whether the denovo assembly method can retrieve the same circular contig from metagenomics DNA. In both packages, the assembly parameters were set to their default values.

To reconstruct the mitogenome of the prey, all metagenomic sequences were assembled denovo in the megahit v.1.2 assembler [19] since this method is particularly suitable for the denovo assembly of complex metagenomic DNA. An implementation of the megahit assembler in MitoZ v.3.4 [20] enables users to filter assemble sequences based on the taxonomic group. The MitoZ assembly pipeline was run with default settings in terms of the k-mer size and other parameters, and arthropods were selected as the target taxonomic group based on previous knowledge of the importance of copepods in the diet of the pipefish [12].

To identify the taxonomic origin and quality of the assembled mitogenomes, the resulting circularised assemblies were blast-searched against the NCBI nucleotide database. These contigs were then annotated using a combination of the MITOS online server [21] and the MitoZ “annotate” command. The phylogenetic placement of the assembled mitogenomes was determined using Bayesian phylogenetic analysis in Beast2 [22]. To this end, mitogenome sequences from 12 closely related pipefish species and four seahorses were identified based on the percentage identity of the blast results and retrieved from the NCBI database. Similarly, the mitogenome of nine closely related copepod species that covered all major taxa within this class were downloaded. A consensus Bayesian phylogenetic tree was constructed for each assembly based on the DNA alignment of 13 protein-coding sequences. The Bayesian phylogenetic site model averaging package bModelTest [18] was used to select the best nucleotide substitution model for each gene. The remaining parameters were set to their default values. Beast2 was run for ten independent replicates, each 100 million iterations long with an initial 33 million burn-in steps. A consensus phylogenetic tree was reconstructed in TreeAnnotator v.1.4, which is part of the Beast2 package and visualised using Figtree v.1.4 (https://github.com/rambaut/figtree).

Since the mitogenomes of the pipefish and its copepod prey were assembled from mitochondrial sequences of an unknown number of predators in the tank, and an unknown number of copepods in their digestive tracts, I tested two methods to quantify the level of genetic diversity in the sequences that formed each assembly. I used the NOVOPlasty heteroplasmy detection pipeline for the pipefish, which identifies the presence of different mitochondria in cells. This method uses a two-step pipeline. In the first step, the circular genome is assembled in the regular way. Then, this assembled contig is used as both the initiation seed and reference template to identify putative variant sites above a user-defined allele frequency threshold.

To identify variable sites in the copepod mitogenome, the raw sequences were first mapped against the assembled mitogenome using BWA v.0.7 [23]. The resulting Sequence Alignment Map (SAM) was converted to its binary BAM format and subsequently sorted in SAMtools v.1.16 [24]. All putative variable sites were identified using Bayesian haplotype variant caller FreeBayes v.1.3 [25] in “pooled-continuous” mode, which is suitable for variant calling in Pool-Seq data when the number of individuals contributing to each pool is unknown. All variant calling parameters were set to their recommended default setting (https://github.com/freebayes/freebayes).

## Limitations

A major limitation of assembling prey mitogenomes from faecal metagenomics datasets is that the number of individuals that contributed to the assembly is unknown. Moreover, when direct observation of the predator and immediate collection of faecal samples are not feasible due to small pellet sizes or logistical constraints, predator faeces may also be a mixture of excretions from an unknown number of individuals, as was the case in this dataset. Further, territorial animals often scent-mark their territories with body excretions near those left behind by other individuals, so faecal samples collected in the field could be a mix of DNA from multiple individuals. As a result, mitogenome assemblies like the ones reported in this dataset can only be considered consensus mitochondrial genomes. However, this level of information is typically adequate for species identification and preliminary surveys of population-level genetic diversity in wild species since the focus of these studies is on monitoring the overall genetic diversity of predator and prey populations and assigning haplotypes to individuals is of less importance.

## Ethics Statement

The research permit for this study (RES2020/101) was granted by the Department of Environment, Forestry and Fisheries of the Republic of South Africa in accordance with IUCN requirements. The animal ethics clearance for this study was approved by the Faculty of Science Ethics Committee at the University of Johannesburg (Ethics Reference Number: 2020-02-06/Teske_Weiss).

## CRediT Author Statement

Not Applicable to a single-authored data note.

## Data Availability

Bioproject (Original data) (NCBI) NCBI nucleotide database (Original data) (NCBI) SRA (Original data) (NCBI) Biosample (Original data) (NCBI)

## Appendix Supplementary materials

Image, application 1
