# Supplementary material for: Reconstruction of the complete mitogenomes of predator and prey from a faecal metagenomic dataset
Source: Data Brief. 2023 Nov 20;52:109830. doi: 10.1016/j.dib.2023.109830 (PMC10698251; doi:10.1016/j.dib.2023.109830)
Supplement: Supplementary file 1 [file mmc1.docx]

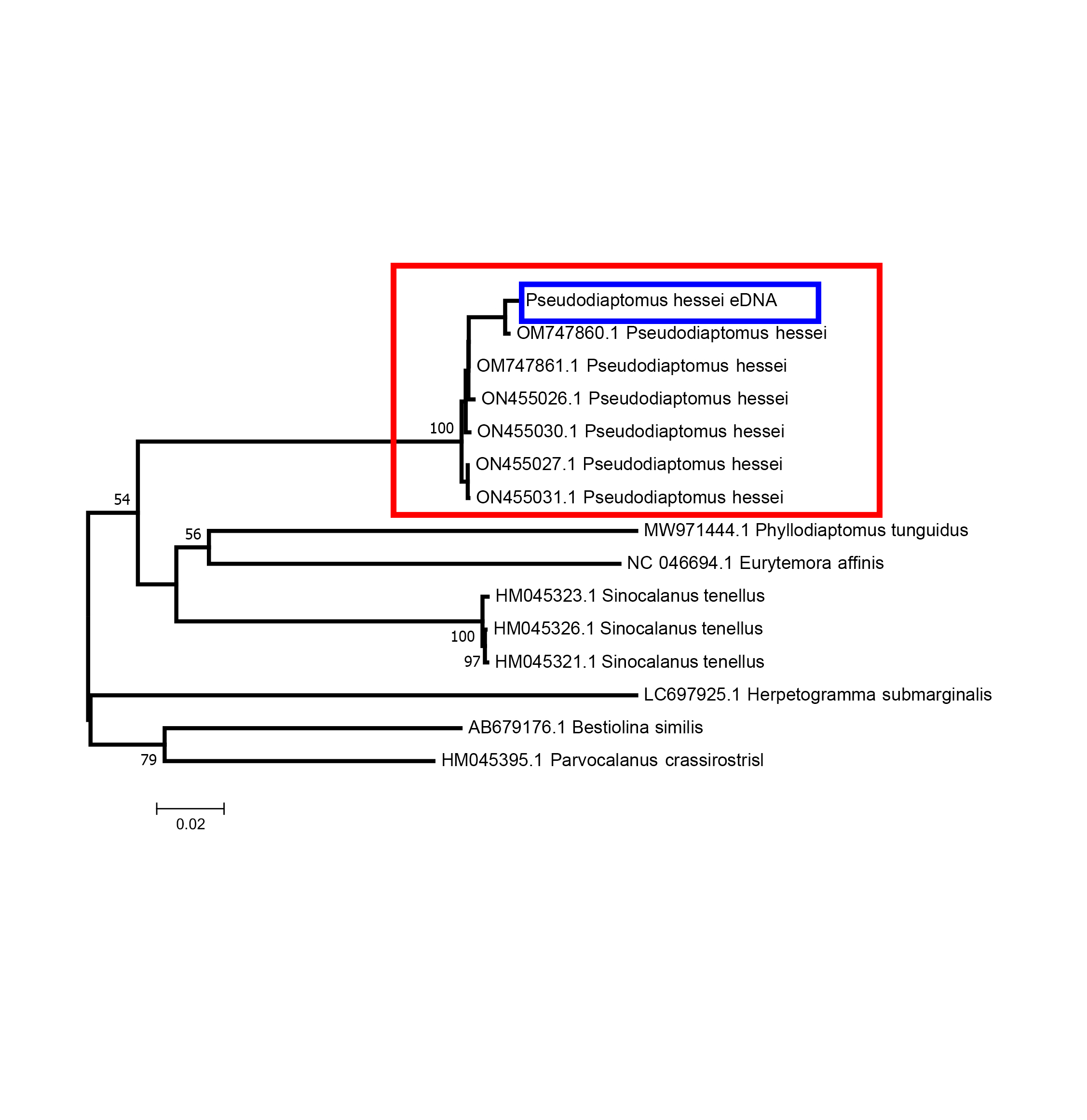


**Figure 1** A Neghborjoing phylogenetic tree based on *COI* sequences of *Pseudodiaptomus hessei* that were assembled from faecal metagenomic DNA, the publicly available *COI* sequences from the same species generated from tissue samples, and those of closely related calanoid copepods. The tree shows that *COI* sequences from the assembled mitogenome (blue rectangle) clustered with other *COI* sequences of the same species that were generated from tissue samples (red rectangle) with 100% bootstrap support.
